# A Longitudinal Study of Gambling Behaviors During the COVID-19 Pandemic in Sweden

**DOI:** 10.3389/fpsyg.2021.708037

**Published:** 2021-10-14

**Authors:** Viktor Månsson, Håkan Wall, Anne H. Berman, Nitya Jayaram-Lindström, Ingvar Rosendahl

**Affiliations:** ^1^Centre for Psychiatry Research, Department of Clinical Neuroscience, Karolinska Institutet and Stockholm Health Care Services, Region Stockholm, Stockholm, Sweden; ^2^Department of Psychology, Uppsala University, Uppsala, Sweden

**Keywords:** gambling, COVID-19, pandemic, worries, high-risk games, problem gambling, longitudinal, pandemic restrictions

## Abstract

This study aimed to investigate changes in gambling behaviors during the first and second waves of the COVID-19 pandemic in Sweden. Participants who had gambled within the past year were recruited from social media and the Swedish National Helpline (*n* = 325, mean age 39.8 years, 64.8% males, 31.3% with problem gambling) and completed an online survey measuring gambling behaviors, consequences of the pandemic in general and worries related to the pandemic. A sub-sample (*n* = 139) completed a follow-up survey, during the second wave. The results showed no significant associations between COVID-19 consequences (financial or increased isolation) and increased monthly gambling behavior. No major migrations were observed between game types. However, gambling on a high-risk game (OR = 7.44, *p* < 0.001) and worrying about mental health due to the pandemic (OR = 2.85, *p* < 0.001) were significantly associated with past year gambling problems and increased monthly gambling problems from the first to the second wave. More longitudinal research is needed in vulnerable populations, to fully understand the long-term consequences of the pandemic.

## Introduction

In the spring of 2020, the Swedish government and health authorities announced recommendations with a goal of limiting the spread of the new coronavirus in society. Citizens were instructed to limit their social contacts and practice physical distancing, leading to increased time spent in solitude and at home. People were encouraged to work from home, where possible, and this has differentially impacted businesses, where several have experienced a financial decline and uncertainty during the pandemic. The changed living conditions and resulting uncertainties during this pandemic have raised concerns about whether it will contribute to an increase in psychiatric symptoms and addictive behaviors (Håkansson et al., 2020; Holmes et al., 2020; Marsden et al., 2020).

Problem gambling (PG) encompasses a continuum of negative financial social and health-related consequences from gambling, with an estimated worldwide past year prevalence between 0.1 and 5.8% (Calado and Griffiths, 2016). Whether the pandemic and its consequences will influence PG is somewhat unclear as there might be effects that can both promote and inhibit gambling. Common high-risk situations for PG are low-stimuli situations, experiencing boredom or lack of structured time (Morasco et al., 2007) and loneliness (Parke et al., 2018), as well as limited opportunities for recreational activities (Hodgins and El-Guebaly, 2004; Pickering et al., 2019) which could potentially promote PG during pandemic times. On the other hand, the pandemic has led to reduced opportunities for gambling. During the first wave in Sweden, major sports events were paused, leading to a period of an almost complete absence of betting objects. In addition, the four state-controlled land-based casinos in Sweden have been closed during the pandemic. However, these represent only a small proportion of gambling since Sweden had already shifted toward mainly online gambling prior to the pandemic. A recent poll showed that 8 out of 10 persons in Sweden who had gambled the previous year, did so at home (Novus, 2020). In addition, in a preventive action in June 2020, the Swedish government implemented a time-limited regulation in the gambling market, which implied limiting the weekly deposit per operator, to 5000 Swedish Krona (1 SEK ∼ 0.1 Euro) at online casinos and electronic gaming machines (EGMs) and restricting bonuses to a maximum of 100 SEK, this was in addition to a mandatory limit-setting on the time spent gambling online. This temporary regulation was set to cease in Dec 31, 2020, but under the continuation of the pandemic it has been extended until November 14th, 2021.

The studies conducted so far during the pandemic on PG, show that the effects are diverse. Pandemic restrictions seem to both promote and reduce gambling, depending on the target population. A review of the emerging data on gambling during the pandemic highlights an overall reduction in gambling behaviors due to the reduced opportunities to gamble but specific sub-groups might increase their gambling, in specific males of young age with prior gambling problems (Hodgins and Stevens, 2021). Studies during the early wave of the pandemic (March to May 2020) point at an overall decline in gambling expenditures and time-spent gambling (Auer et al., 2020; Gainsbury et al., 2020; Lindner et al., 2020; Auer and Griffiths, 2021). Operators with Swedish licenses indicated an increase in number of online casino gamblers, but significant decrease in number of high-risk players and decrease in mean average daily bets (Auer and Griffiths, 2021). An expected drop in sports betting was reported during this phase, with only a slight increase in online casino gambling, which was not in proportion to the reduction in sports betting (Lindner et al., 2020).

Whether spending more time at home is a high-risk factor for increased gambling, is at present unclear (Håkansson, 2020). This might be due to the fact that being at home could reflect a range of underlying circumstances, such an enforced quarantine, or because of job termination, temporary lay-off or working remotely. A study from the United Kingdom found that levels of stress, depression and anxiety increased during the first phase of the pandemic for both non-gamblers and individuals with Problem Gambling (IPGs), with no differences between the groups, although IPGs reported higher baseline ratings of mental health problems (Sharman et al., 2021). Furthermore, a Canadian study during the pandemic showed that high-risk and online gambling was associated with higher ratings of anxiety and depression (Price, 2020).

A Swedish study found that past-month gambling during the pandemic had a stronger association with PG compared to past year gambling for several game types (i.e., any sports betting, land-based casinos, and EGMs); however, for online horse betting, an opposite association was found (Håkansson, 2020). This is in line with previous research pointing toward an important indicator of PG which is frequent gambling on high-risk games, such as EGMs and online casinos, a relationship that is well established (Binde et al., 2017; Wall et al., 2020). These high-risk games are characterized by short intervals between bet and outcome and when a new bet can be placed, which provides almost unlimited gambling opportunities (Griffiths and Auer, 2013).

The temporary reduction in conventional sport betting events and the limitations on everyday lives, can be described as a naturalistic experiment. Due to different life circumstances, adherence to recommendations differs in the population, e.g., by type of occupations, where in some cases working remotely is not an option. Despite the growing knowledge on the impact of the pandemic on PG, several important questions remain to be addressed. Firstly, is the pandemic’s impact on people’s lives and well-being associated with increased gambling behaviors? Secondly, to what extent did the temporary cessation of sports betting trigger a migration into high-risk games associated with PG? Previous studies during the pandemic have focused either on gambling operator’s data or estimations based on cross-sectional designs. Given this unprecedented situation in modern times, it is important to explore pandemic effects on addictive behaviors such as gambling via reports from individuals who gambles on a regular basis and by individuals with PG.

The present study therefore builds upon the existing knowledge and addresses the limitations, by examining the impact of the COVID-19 pandemic on gambling behaviors and PG using a longitudinal design over the first two waves of the pandemic. This using a sample recruited via social media and a national gambling helpline, including individuals living in Sweden who reported gambling during the past year. The specific aims were to investigate the associations between:

– COVID-19 consequences (worries, personal finances, and increased social isolation) and gambling behaviors and PG.
– Migrations between type of games, in particular from sports betting into high-risk games and PG.

## Materials and Methods

### Recruitment

This study was initiated in April 2020. Participants were recruited mainly through Facebook, but also Twitter and advertisement on the homepage for the Swedish National Helpline for IPGs (Stödlinjen). The advertisement targeted individuals who had gambled during the past year. Facebook ads targeted users who had shown previous interest in gambling related topics, such as poker, live-betting, casino, roulette, bingo, sports-betting and black-jack. Recruitment took place between May 5 and October 31, 2020 and data were collected using the SurveyXact online survey tool (Ramböll, 2018).

All participants provided their informed consent regarding participating in research and handling of personal data and were asked for consent to be contacted for a follow up questionnaire regarding their gambling. Given the uncertainty of the pandemic, the time point for the follow-up survey was not decided at the inclusion. An email with a link to the follow-up survey was sent out on November 23, 2020. The survey did not use forced answers, since these type of answers might have negative effect on data quality (Sischka et al., 2020). Participants were sent up to six reminders via e-mail and were given until the end of December 2020 to complete follow-up. No remuneration was given for participation.

### Measures

The study utilizes three measure points. February 2020 was chosen as a retrospective baseline of gambling behaviors prior to the pandemic in Sweden. The measure points and variables at each point were:

*February 2020* (retrospective baseline) consisted of measures on (1) gambling behavior: type of game, frequency and expenditures and (2) self-rated gambling problems during February 2020.

*The first-wave* online survey consisted of: (1) demographic characteristics; (2) current restrictions and consequences due to the pandemic; (3) *gambling behaviors* during the previous month; (4) *worries* about financial, mental and physical well-being related to the pandemic (COVID-19 worries); (5) Problem Gambling Severity Index (PGSI); (6) self-rated gambling problems the previous month; and (7) status of self-exclusion from gambling.

*The second-wave* survey consisted of: (1) current restrictions and consequences due to the pandemic; (2) *gambling behaviors* during the previous month; (3) *worries* about financial, mental and physical well-being related to the pandemic: (4) self-rated gambling problems the previous month; and (5) status of self-exclusion from gambling.

*Restrictions and consequences* due to the pandemic were assessed through a list of possible consequences that might affect everyday lives rated as yes or no answers whether the participants had experienced the consequence or not. These included financial (e.g., bankruptcy, lay-offs), health-related (e.g., COVID-19 infection) for the participant and/or peers, and social isolation consequences such as being in quarantine due to high-risk group, working or studying from home or home schooling of children. *Gambling behavior* was measured by presenting a list of games where participants were asked to report all games played the previous month, how frequent each game was played during that month (ranging from 1 = monthly to 6 = several times per day) and how much money (in SEK) was spent monthly on each game.

*Worries* concerning health (physical and mental) and private finances due to the COVID-19 pandemic were rated on a four-point scale from 0 = *no, not at all* to 3 = *yes, very much*, with items such as: *Have you, due to the pandemic, been worried about your physical health during the last month?*

Problem gambling was rated with the PGSI, a nine-item instrument with a total score ranging from 0 to 27, assessing the presence of PG during the previous 12 months (Wynne and Ferris, 2001). In order to capture changes in PG-status, a single item measured gambling problem during the prior month; *Have you had problems with gambling the last month?* on a four-point Likert-scale, ranging from *not at all* to *extreme*. Self-exclusion from gambling was measured by participants stating whether they were registered at the National Self Exclusion Register^1^ with the alternatives: not registered, 1, 3, or 6 months or until further notice). This self-exclusion register is a part of the Swedish Gambling Act, a legislative licensed gambling market introduced in January 2019.

### Operationalization of Raw Data

Based on previous research, online slots, online live-betting and EGMs were defined as game types associated with increased high-risk of PG, further on referred to as *high-risk games* (Griffiths and Auer, 2013; Binde et al., 2017; Lopez-Gonzalez et al., 2019; Wall et al., 2020). One variable regarding high-risk games was constructed: *any high-risk game*, the variable was coded as “1” if a participant reported gambling on a high-risk game and “0” if not. Increased gambling frequency was analyzed as the difference of the highest gambling frequency on any game type between the current and the previous timepoints. The variable was coded as “1” if an individual had increased gambling frequency compared to the previous timepoint and “0” if not. Eleven variables regarding COVID-19 restrictions were collapsed into two, one variable related to negative financial consequences, defined as having experienced at least one of the following: being laid-off, company reconstruction, bankruptcy or notice of job termination. A second variable related to increased social isolation during the pandemic was defined as having experienced at least one of the following: self-quarantine due to infection or high-risk group, working or studying from home and/or living with someone infected with the virus. A cut off of ≥5 on the PGSI was used for classification as an IPG, a threshold that has been suggested to improve the classification accuracy of PG (Williams and Volberg, 2014; Binde et al., 2017). The outcome variable of past month gambling problems was analyzed as a binary variable where “0” represented no gambling problems and “1” any gambling problem. Finally, age was centered around its mean.

### Statistical Methods

Past year gambling problems, gambling problems and gambling frequency during the first wave were analyzed using generalized linear models (GLM) with a binominal link function. Binary longitudinal data was analyzed using generalized linear mixed-effects models (GLMMs). Restrictions due to COVID-19, gambling on a high risk game and worries due to COVID-19 were added as time-varying covariates in the longitudinal models. The R-package lme4 (Bates et al., 2014) was used to fit the longitudinal models. Both models analyzing past month gambling problems were adjusted for the pre-pandemic level of gambling problems.

## Results

The sample consisted of 325 participants recruited between May 5 and October 31, 2020, of these, 283 reported type of game prior to the pandemic and 267 reported type of game during the first wave. A subsample of 139 participants completed the second wave survey from November 1 and onward, or at least 1 month after the first assessment. The mean time between the first and second assessments was 133 days (SD = 55). Among those who participated, a majority were males (64.8%) and the mean age was 39.8 years (SD = 14.3). Most of the participants were employed (62.5%) and 31.3% were classified as IPGs according to PGSI during the first wave. Those who completed the second survey did not differ in sex, civil status or age compared to those who only filled out the first survey but had lower mean PGSI scores (4.6 compared to 6.0). Among those who reported being self-excluded (*n* = 39) from gambling at the first wave, 31 (80%) reported past year gambling problems, and during the second wave 9 (56%) reported gambling problems and 11 (61%) reported any gambling the previous month despite being self-excluded from gambling. See Table 1 for demographics and COVID-19-related variables and Table 2 for gambling-related variables, with proportions presented relative to the total number of participants responding to item.

**TABLE 1:** Sample characteristics and COVID-19 related variables for the first and second waves.

Variable	First wave (*N* = 325)	Second wave (*N* = 139)
**Gender, *n* (%)**		
Women	101 (33.6)	48 (33.6)
yMen	195 (64.8)	94 (65.7)
Prefer not to say	5 (1.7)	1 (0.7)
Age, mean (sd)	39.8 (14.3)	40.5 (14.1)
Income (SEK), median (IQR*)	21,000 (18,768)	22,000 (19,300)
**Civil status, *n* (%)**		
Single	109 (37.6)	55 (40.6)
In a relationship	173 (59.7)	81 (58.7)
Other	8 (2.8)	4 (0.7)
Participants with children, *n* (%)	90 (37.8)	44 (36.4)
**Occupation**		
Student	41 (13.6)	
Unemployed	17 (5.7)	
Employed	188 (62.5)	
Own company	39 (13.0)	
Other (retired)	16 (5.3)	
**Type of COVID-19 consequences (%)**		
**Social isolation and infection**		
Home office	92 (32.4)	51 (37.8)
Taking part in distance education	45 (15.9)	20 (14.8)
Quarantine due to high-risk group	39 (13.7)	15 (11.1)
Self-quarantine due to COVID-19 infection	23 (8.1)	7 (5.2)
Family member infected by COVID-19	21 (7.4)	6 (4.4)
Infected by COVID-19	17 (6.0)	3 (2.2)
Home-schooling of children	8 (2.8)	3 (2.2)
**Financial consequences**		
Short term lay-off	42 (14.8)	8 (5.9)
Short term lay-off for family member	29 (10.2)	6 (4.4)
Notice of job termination	15 (5.3)	5 (3.7)
Family member receiving job notice	12 (4.2)	1 (0.7)
Company reconstruction	5 (1.8)	1 (0.7)
Bankruptcy	2 (0.7)	0 (0)
No consequences	76 (26.8)	41 (30.4)
**Worried about personal finances due to the pandemic, *n* (%)**		
No, not at all	140 (48.3)	76 (55.5)
Yes, some	103 (35.5)	37 (27.0)
Yes, quite a lot	30 (10.3)	12 (8.8)
Yes, very much	17 (5.9)	12 (8.8)
**Worried about physical health due to the pandemic, *n* (%)**		
No, not at all	131 (46.3)	55 (40.1)
Yes, some	101 (35.7)	50 (36.5)
Yes, quite a lot	39 (13.8)	24 (17.5)
Yes, very much	12 (4.2)	8 (5.8)
**Worried about mental health due to the pandemic, *n* (%)**		
No, not at all	151 (53.0)	64 (46.7)
Yes, some	84 (29.5)	43 (31.4)
Yes, quite a lot	38 (13.8)	22 (16.1)
Yes, very much	12 (4.2)	8 (5.8)

*IQR = Interquartile Range.

**TABLE 2:** Gambling-related variables.

	Pre-pandemic (*n* = 283)	First wave (*n* = 267)	Second wave (*n* = 137)
Money spent on gambling (SEK), median (IQR)	195 (2,000)	90 (3,000)	0 (200)
PGSI 5+, *n* (%)		79 (31.3)	31 (25.0)
Self-excluded (via *Spelpaus.se*)		39 (14.7)	25 (18.8)
**Gambling problems past month, *n* (%)**			
Not at all	200 (76.0)	189 (71.3)	104 (78.8)
Some	35 (13.3)	22 (8.3)	11 (8.3)
Quite a lot	16 (6.1)	18 (6.8)	8 (6.1)
To a large extent	12 (4.6)	36 (13.6)	9 (6.8)
Number of games played, mean (sd)	2.04 (2.33)	1.65 (2.07)	1.90 (2.33)

### Pandemic Restrictions and Consequences

During the first wave, 86.3% of the sample reported having consequences of increased social isolation, with working from home being the most common during both waves, 32.4 and 37.8%, respectively. More than a third (37%) reported financial consequences, with short term lay off as the most common, reported by 14.8% (see Table 1 for details). Neither social isolation nor financial consequences were associated with increased gambling problems or gambling frequency (see Table 3 and Table 4).

**TABLE 3:** Logistic regression model output for first wave.

	PGSI 5^+^	Increased gambling frequency	Gambling problems
	Estimate [95% CI]	Estimate [95% CI]	Estimate [95% CI]
Intercept	0.11** [0.02–0.50]	0.15** [0.04–0.57]	0.02*** [0–0.14]
Age	0.98 [0.95–1.01]	0.99 [0.97–1.01]	1.01 [0.97–1.05]
Male	1.94 [0.86–4.58]	0.72 [0.35–1.46]	0.94 [0.30–3.03]
Isolation due to COVID-19	0.42* [0.20–0.87]	1.76 [0.90–3.60]	0.51 [0.18–1.45]
Economic consequences due to COVID-19	0.68 [0.28–1.56]	1.03 [0.47–2.17]	0.75 [0.21–2.42]
Any high-risk game	7.44*** [3.57–16.53]	2.92** [1.50–5.86]	8.43*** [2.92–0.27.99]
Worry about finances	1.6 [0.97–2.68]	1.14 [0.73–1.75]	1.65 [0.83–3.33]
Worry physical health	1.07 [0.63–1.79]	0.80 [0.51–1.24]	1.15 [0.55–2.35]
Worry mental health	2.85*** [1.70–5.02]	1.62* [1.04–2.53]	1.49 [0.68–2.92]
Gambling problems pre-COVID-19	–	–	31.68*** [10.17–120.57]

Estimates in odds ratios (OR). *p ≤ 0.05, **p < 0.01, ***p < 0.001. Bold values indicates a significant association.

**TABLE 4:** Logistic regression model output for change in gambling frequency and gambling problems from the first to the second wave.

	Increased gambling frequency	Gambling problems
	Estimate [95% CI]	Estimate [95% CI]
Intercept	0.023 [0.003–0.18]**	0.17 [0.01–4.14]
Time	2.03 [1.08–3.82]*	0.46 [0.16–1.34]
Age	1.00 [0.98–1.02]	0.98 [0.94–1.02]
Male	1.80 [0.86–3.82]	0.70 [0.21–2.33]
Isolation due to COVID-19	1.05 [0.56–1.95]	0.59 [0.21–1.65]
Economic consequences due to COVID-19	1.27 [0.56–2.86]	0.86 [0.24–3.15]
Any high-risk game	2.36 [1.28–4.39]*	9.57 [3.08–29.75]***
Worry about finances	0.76 [0.49–1.17]	1.68 [0.81–3.46]
Worry physical health	1.06 [0.68–1.65]	0.96 [0.47–1.94]
Worry mental health	1.40 [0.90–2.18]	2.82 [1.42–5.64]**
Gambling problems pre-COVID-19	–	15.84 [4.82–52.06]***

Estimates in odds ratios (OR). *p ≤ 0.05, **p < 0.01, ***p < 0.001. Bold values indicates a significant association.

### Game Types and Expenditures

The most common type of game played was online casino slots during the pre-pandemic measurement and during the first wave (33.9 and 34.8%, respectively), whereas online odds games were the most common game type during the second wave of the pandemic, 35%, see Supplementary Table 1 for information on game types played among the participants. Two sports bettors added online casino during the first wave of the pandemic, but none migrated from sports betting to online casino games. Between the first and second wave, none migrated from sports betting to online casino. Among those who had engaged in online casino games in February 2020 (*n* = 103), 84 reported continued online casino gambling during the first wave of the pandemic, and of those included in the second wave, 31 of 37 reported continued online casino gambling. The patterns among those who wagered on sports events were similar, where 48 of 77 continued during the first wave and 28 of 32 continued betting on sports during the second wave. Further, 12 individuals who stopped sports betting during the first wave returned to sports betting during the second wave.

During the first wave of the pandemic, 43.5% of the participants reported no changes in gambling expenditures, 31.9% reported increased expenditures and 24.6% decreased gambling expenditures compared to the pre-pandemic timepoint. Those who increased their gambling expenditures (65% males, mean age = 39) also reported higher mean PGSI scores compared to those who decreased (84% males, mean age = 38) or maintained expenditures (59% males, mean age = 42), 9.7 PGSI points compared to 2.8 and 2.9, respectively.

Among the participant (*n* = 139) who provided a follow up measurement during the second wave, 29.5% reported unchanged expenditures, 31.1% increased expenditures, and 39.3% reported decreased expenditures compared to the first wave. Those who reported increased gambling expenditures were to a greater extent males (76%) compared to those who decreased their gambling expenditures (63%) and reported lower mean PGSI scores, 2.3 compared to 8.2. Participants who reported unchanged gambling expenditures (60% males) reported the lowest mean PGSI scores, 1.8.

#### High-Risk Games

During the first wave of the pandemic, 15 (5.4%) individuals reported that they had started a high-risk game, 116 (41.4%) reported they had continued, and 20 (6.1%) individuals reported that they had stopped gambling on a high-risk game compared to the pre-pandemic timepoint. During the second wave of the pandemic, 9 (4.8%) started, 42 (18.8%) continued, and 16 (7.6%) individuals stopped gambling on a high-risk game compared to the first wave.

#### Problem Gambling and Gambling Frequency During the First Wave

All models were tested for overdispersion. The model analyzing gambling problems the previous month had a dispersion parameter which differed from one. This model was analyzed with a quasibinomial link to compensate for the overdispersion. We found that gambling on a high-risk game (OR = 7.4, *p* < 0.001) and worrying about mental health (OR = 2.85, *p* < 0.001) were associated with increased odds of experiencing past year’s gambling problems (PGSI ≥ 5) whereas social isolation due to COVID-19 was associated with 58% lower odds (OR = 0.42, *p* = 0.02) of past year’s gambling problems. Gambling on a high-risk game was associated both with increased odds of experiencing past month gambling problems (OR = 8.43, *p* < 0.01) and increased gambling frequency (OR = 2.92, *p* < 0.01) during the first wave. Furthermore, worrying about mental health during COVID-19 was associated with increased gambling frequency (OR = 1.62, *p* = 0.033) during the first wave. See Table 3 for further details.

#### Development of Problem Gambling and Gambling Frequency Between the First and Second Wave

**FIGURE 1:**
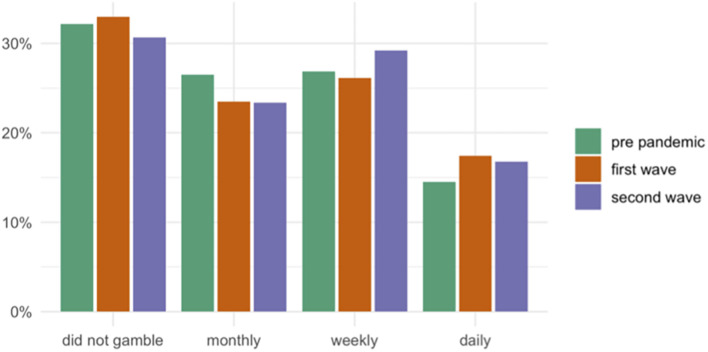
Highest gambling frequency in a game type per time period. Pre-pandemic *N* = 283, first wave *N* = 267 and second wave *N* = 137.

Both longitudinal models were tested with and without random slopes, and since the addition of random slopes did not improve the models, we chose to present the results from the more parsimonious models with random intercepts only. We found a main effect of time (OR = 2.04, *p* = 0.028) for increased gambling frequency between the first and second wave but not for gambling problems. Further, gambling on a high-risk game was associated with both increased gambling frequency (OR = 2.37, *p* < 0.01) and gambling problems (OR = 9.57, *p* < 0.0001) at any timepoint. We also found that worrying about mental health due to COVID-19 was associated with increased odds of experiencing gambling problems at any timepoint (OR = 2.83, *p* = 0.01). See Table 4 for all model estimates and Figure 1 for gambling frequency at the different timepoints.

## Discussion

The results from this longitudinal study showed no associations between COVID-19 related consequences and increased gambling behavior and no substantial migrations from sports betting to high-risk games. Worries about mental health due to the pandemic were associated with increased odds of experiencing gambling problems both over the past 12 months, increased risk of gambling problems between the two measure points and increased gambling frequency in the first wave. Noteworthy is that the type of games played among the study participants were stable between the three timepoints, which indicates distinct and unchanged preferences.

Importantly, gambling on high-risk games was the strongest predictor of gambling problems and increased gambling frequency both during the first and second wave of the pandemic. These findings support previous research which shows that certain game types, such as online casino games and EGMs, are associated with PG (Binde et al., 2017; Wall et al., 2020). Further, a recent study from the United Kingdom found that the vast majority stopped or reduced their gambling involvement during lockdown, but among men who switched game types, PG was more common (Sharman et al., 2021). This suggests that online gambling operators should pay particular attention to customers who gamble on high-risk game and have shown problematic gambling behaviors in the past. Among those gambling on high-risk games there might also be a higher proportion of unlicensed gambling, since these operators typically offer online casino games without legislated consumer protection of bonuses and deposit limits. The concern of migration to high-risk game after cancelation of sports event was not supported, perhaps an effect of the new temporary restrictive legislations on the Swedish gambling.

As noted in previous studies during the pandemic (Håkansson, 2020), special attention is needed toward vulnerable individuals, in this study reflected by the link between increased worry about mental health due to the pandemic and self-reported gambling problems. This shows that the same individuals vulnerable to PG are also more likely to experience negative impact on their mental health due to the pandemic. The high proportion of co-occurring mental health problems among IPGs is well documented (Lorains et al., 2011; Dowling et al., 2015). Population samples of IPGs have reported high levels of psychiatric comorbidity with more than one third meet criteria for an anxiety disorder (Lorains et al., 2011). A Norwegian study during the pandemic showed that those with prior mental health problems, financial problems and those living alone were also more likely to experience higher levels of pandemic related worries (Blix et al., 2021). The effect of the pandemic is diverse as noted but might have an amplifying affect on previous risk factors such a symptoms of anxiety. In their Pathways model, Blaszczynski and Nower (2002) describes the emotional vulnerable type of gambler, a subtype that gambles primarily to alleviate symptoms of depression and anxiety. Moreover, studies have shown that IPGs more commonly report difficulties in emotion regulation strategies, namely, acceptance of emotional states, low impulse control and difficulties in maintaining goal directed behavior in the presence of negative emotional states (Velotti et al., 2021). The direction of the association between worries for mental health and PG found in the present study is unclear, but on a speculative note their might be participants gambling to distract from worries, including pandemic related ones. On the other hand, having experienced negative consequences from gambling within the previous year, reflected by the PGSI-measure in the present study, may be a reason for increased worries of the pandemics’ impact on mental health.

COVID19-related restrictions on everyday life were not linked to increased monthly gambling behavior among the participants. Physical distancing, working from home and self-quarantine are limitations on everyday social contacts and recreational activities that might exacerbate depressive symptoms. On the other hand, spending more time at home with family, might also inhibit gambling. In a sample (*n* = 135) of individuals with PG in Italy, the initial lockdown seemed to have reduced gambling problems at least in the short term. The authors suggest that an increase in social control within families during isolation, could have served as a protective factor (Donati et al., 2021). A potential confounder in relation to increased time at home that needs to be addressed is that working from home is less common among socioeconomic vulnerable groups.

A recent study showed that PG was more common among those being furloughed and among woman reporting being in quarantine due to the pandemic (Wardle et al., 2021). Apart from those circumstances, there were no associations between COVID-19 personal and financial consequences and PG, as noted in the present study. The effects of financial difficulties due to the pandemic on PG might be delayed and remains to be better understood in Sweden and globally. In the 3 years following the financial crises in Iceland, an increase in gambling behavior occurred, and an association was identified between financial difficulties and increased expenditures on lotto and scratch card tickets; also, there was an increase of interest in gambling among individual with no prior gambling experience (Olason et al., 2017). Throughout the pandemic, the Swedish government has released emergency economic relief packages, which perhaps dampened the negative short-term financial effects for many businesses and their employees.

Another important tool in reducing PG is the national self-exclusion register (Spelpaus). The reason why people choose to utilize the national self-exclusion register has not yet been investigated, but an obvious assumption is that many self-exclude as way to reduce harm from gambling. The large proportion of IPGs (80%) among those that were registered for self-exclusion in the present study gives strength to this assumption. Nevertheless, self-exclusion should have a inhibiting effect on future gambling behavior, but a fairly large proportion (61%) among those self-excluded reported gambling at follow up. An explanation may be that IPGs self-exclude from gambling within the licensed market but might continue gambling on online platforms outside of the licensed market. A recent study found that among those self-excluded, 38 percent reported gambling despite the self-exclusion, and commonly on online casinos (Håkansson and Widinghoff, 2020).

This study utilizes self-reports and caution is needed when interpreting gambling expenditures. Even though studies shows that self-reports correlate with actual losses from operator data, higher expenditures are harder to estimate and might be susceptible to distortions (Braverman et al., 2014; Auer and Griffiths, 2017). Moreover, wordings of items in a survey might allow for different interpretations (Wood and Williams, 2007). Some responses in the present study indicated that participants might have reported their revenue and not money transferred into gambling. The survey did not specify how to respond when gambling with previous wins, i.e., participants might have reported zero expenditures due to previous wins, while others might have included previous wins in their estimation.

### Strengths and Limitations

A strength of the study is its longitudinal design which covered 2 critical phases of the COVID-19 pandemic and the inclusion of a sample of IPGs (31.3% in the first wave) and individuals playing online slots (33.9%). Even though research studies relying on self-reports have their limitations, subjectively experienced worries and impact on an individual’s life constitute important data that cannot be captured from gambling operators’ data. Nonetheless, some limitations of the current study need to be addressed. One concerns the sample size, specifically at follow up. The sub-sample agreed to contribute to follow-up data consisted of a smaller proportion of IPGs (25% vs. 31.3%) which might have skewed the results toward an underestimation of gambling behavior at follow-up. Yet, there is an absence of longitudinal studies in this population at present, and the circumstances specific to pandemic-related studies need to be taken into consideration. In an ambition to reduce the potential self-selection bias, i.e., that individuals more prone to have experienced a recent change in their status of PG would be more likely to join the study, the invitation to participate did not mention “gambling problems” or any negative effects from gambling, but targeted people who gambled on a regular basis. Nevertheless, we cannot disregard the risk of collider bias, i.e., that having recently developed gambling problems and gambled on online casinos where the Swedish National Helplines’ website is linked, might influence individuals to enroll in an online survey on gambling and be included in the present sample. At the same time, the results do not point in that direction since the high-risk factors which were identified in the present study, are in line with previous studies based on samples from the general population (Public Health Agency, 2019; Auer et al., 2020) and on samples of IPGs (e.g., Ronzitti et al., 2018; Wall et al., 2020).

### The Post-COVID19-Era in Gambling and Future Studies

The long-term effects of the pandemic on gambling behavior are yet to be understood. Overall gambling participation and land-based gambling among IPGs decreased initially during the pandemic (Auer et al., 2020; Gainsbury et al., 2020; Lindner et al., 2020; Donati et al., 2021; Hodgins and Stevens, 2021) and there was a temporary reduction in sports betting (Wardle et al., 2021) and a small proportion of IPGs increased their gambling (Håkansson, 2020). As IPGs are known to seldom seek treatment (Sharman et al., 2019), reduced opportunities for taking part in face-to-face treatment and self-help groups might have had a negative effect on a smaller proportion of IPGs who are help- and/or treatment-seeking. On the other hand, the pandemic has rapidly highlighted the importance of telemedicine and the need for adapting treatments without face-to-face meetings, providing important experiences for post-pandemic times. Future studies should focus on populations experiencing long-term negative financial effects of the pandemic and close monitoring of those initiating high-risk games during a pandemic seems warranted. Additionally, investigating transitions between addictive behaviors during the pandemic is of importance.

## Conclusion

The current longitudinal study demonstrated that COVID-19 related consequences were not associated with increased gambling and gambling problems during the first or second phase of the pandemic in Sweden, with few migrations from sports betting to high-risk games. Being worried about mental health due to the pandemic was linked to gambling problems. Finally, gambling on a high-risk game during the pandemic was the most important indicator of PG.

## Data Availability Statement

The R-code that supports these analyses can be found at github.com/hakanwall/covid_study. The data for this study are available upon request without personal details such as age, gender and income. This, however, affects the reproducibility of the analyses.

## Ethics Statement

The study was conducted according to the guidelines of the Declaration of Helsinki and approved by the Swedish Ethical Review Authority (ref. nr. Dnr 2020-01809). The participants provided an online informed consent to participate in this study. Software should be listed seperately: Software: R version 3.6.3.

## Author Contributions

HW, VM, AB, NJ-L, and IR: conceptualization and writing–reviewing and editing. VM and HW: methodology and writing–original draft preparation. IR: software, R version 6.2., and validation. HW: formal analysis. IR and HW: data curation. NJ-L: funding acquisition. All authors have read and agreed to the published version of the manuscript.

## Conflict of Interest

VM has received a research grant for another study from the Svenska Spel’s Independent Research Council, financed by the state-owned gambling company Svenska Spel AB. AB is a board member of the Svenska Spel’s Independent Research Council. The remaining authors declare that the research was conducted in the absence of any commercial or financial relationships that could be construed as a potential conflict of interest.

## Publisher’s Note

All claims expressed in this article are solely those of the authors and do not necessarily represent those of their affiliated organizations, or those of the publisher, the editors and the reviewers. Any product that may be evaluated in this article, or claim that may be made by its manufacturer, is not guaranteed or endorsed by the publisher.

## Footnotes

1
https://www.spelpaus.se/

## References

[B1] Auer M. Griffiths M. D. (2017). Self-Reported losses versus actual losses in online gambling: an empirical study. *J. Gambling Stud*, 33 795–806. 10.1007/s10899-016-9648-0 27815667 PMC5579145

[B2] Auer M. Griffiths M. D. (2021). Gambling before and during the COVID-19 pandemic among online casino gamblers: an empirical study using behavioral tracking data. *Int. J. Ment. Health Addict.* 1–11. 10.1007/s11469-020-00462-2 33551689 PMC7852466

[B3] Auer M. Malischnig D. Griffiths M. D. (2020). Gambling before and during the COVID-19 pandemic among european regular sports bettors: an empirical study using behavioral tracking data. *Int. J. Ment. Health Addict. [Online ahead of print]* 1–8. 10.1007/s11469-020-00327-8 32837423 PMC7259431

[B4] Bates D. Machler M. Bolker B. Walker S. (2014). Fitting linear mixed-effects models using lme4. *J. Stat. Softw.* 67 1–48.

[B5] Binde P. Romild U. Volberg R. A. (2017). Forms of gambling, gambling involvement and problem gambling: evidence from a Swedish population survey. *Int. Gambling Stud.* 17 490–507. 10.1080/14459795.2017.1360928

[B6] Blaszczynski A. Nower L. (2002). A pathways model of problem and pathological gambling. *Addiction* 97 487–499. 10.1046/j.1360-0443.2002.00015.x 12033650

[B7] Blix I. Birkeland M. S. Thoresen S. (2021). Worry and mental health in the Covid-19 pandemic: vulnerability factors in the general Norwegian population. *BMC Public Health* 21:928. 10.1186/s12889-021-10927-1 34001071 PMC8127278

[B8] Braverman J. Tom M. A. Shaffer H. J. (2014). Accuracy of self-reported versus actual online gambling wins and losses. *Psychol. Assessment* 26 865–877. 10.1037/a0036428 24708074

[B9] Calado F. Griffiths M. D. (2016). Problem gambling worldwide: an update and systematic review of empirical research (2000-2015). *J. Behav. Addict.* 5 592–613. 10.1556/2006.5.2016.073 27784180 PMC5370365

[B10] Donati M. A. Cabrini S. Capitanucci D. Primi C. Smaniotto R. Avanzi M. (2021). Being a gambler during the COVID-19 pandemic: a study with Italian patients and the effects of reduced exposition. *Int. J. Environ. Res. Public Health* 18:424. 10.3390/ijerph18020424 33430353 PMC7825745

[B11] Dowling N. A. Cowlishaw S. Jackson A. C. Merkouris S. S. Francis K. L. Christensen D. R. (2015). Prevalence of psychiatric co-morbidity in treatment-seeking problem gamblers: a systematic review and meta-analysis. *Aust. N. Z. J. Psychiatry* 49 519–539. 10.1177/0004867415575774 25735959 PMC4438101

[B12] Gainsbury S. M. Swanton T. B. Burgess M. T. Blaszczynski A. (2020). Impacts of the COVID-19 shutdown on gambling patterns in australia: consideration of problem gambling and psychological distress. *J. Addict. Med. [Online ahead of print]* 10.1097/ADM.0000000000000793 33323696 PMC8562923

[B13] Griffiths M. Auer M. (2013). The irrelevancy of game-type in the acquisition, development, and maintenance of problem gambling. *Front. Psychol.* 3:621. 10.3389/fpsyg.2012.00621 23335910 PMC3547280

[B14] Håkansson A. (2020). Changes in gambling behavior during the COVID-19 pandemic-a web survey study in Sweden. *Int. J. Environ. Res. Public Health* 17:4013. 10.3390/ijerph17114013 32516880 PMC7312016

[B15] Håkansson A. Fernández-Aranda F. Menchón J. M. Potenza M. N. Jiménez-Murcia S. (2020). Gambling during the COVID-19 crisis - a cause for concern. *J. Addict. Med.* 14 e10–e12. 32433365 10.1097/ADM.0000000000000690 PMC7273946

[B16] Håkansson A. Widinghoff C. (2020). Gambling despite nationwide self-exclusion-a survey in online gamblers in Sweden. *Front. Psychiatry* 11:599967. 10.3389/fpsyt.2020.599967 33343428 PMC7738608

[B17] Hodgins D. C. El-Guebaly N. (2004). Retrospective and prospective reports of precipitants to relapse in pathological gambling. *J. Consult. Clin. Psychol.* 72 72–80. 10.1037/0022-006x.72.1.72 14756616

[B18] Hodgins D. C. Stevens R. M. G. (2021). The impact of COVID-19 on gambling and gambling disorder: emerging data. *Curr. Opin. Psychiatry*. 34 332–343. 10.1097/yco.0000000000000709 33859126 PMC8183251

[B19] Holmes E. A. O’connor R. C. Perry V. H. Tracey I. Wessely S. Arseneault L. (2020). Multidisciplinary research priorities for the COVID-19 pandemic: a call for action for mental health science. *Lancet Psychiatry* 7 547–560. 32304649 10.1016/S2215-0366(20)30168-1 PMC7159850

[B20] Lindner P. Forsström D. Jonsson J. Berman A. H. Carlbring P. (2020). Transitioning between online gambling modalities and decrease in total gambling activity, but no indication of increase in problematic online gambling intensity during the first phase of the COVID-19 outbreak in sweden: a time series forecast study. *Front Public Health* 8:554542. 10.3389/fpubh.2020.554542 33117770 PMC7550730

[B21] Lopez-Gonzalez H. Estévez A. Griffiths M. D. (2019). Internet-Based structural characteristics of sports betting and problem gambling severity: is there a relationship? *Int. J. Ment. Health Addict.* 17 1360–1373. 10.1007/s11469-018-9876-x

[B22] Lorains F. K. Cowlishaw S. Thomas S. A. (2011). Prevalence of comorbid disorders in problem and pathological gambling: systematic review and meta-analysis of population surveys. *Addiction* 106 490–498. 21210880 10.1111/j.1360-0443.2010.03300.x

[B23] Marsden J. Darke S. Hall W. Hickman M. Holmes J. Humphreys K. (2020). Mitigating and learning from the impact of COVID-19 infection on addictive disorders. *Addiction* 115 1007–1010. 10.1111/add.15080 32250482 PMC9364227

[B24] Morasco B. J. Weinstock J. Ledgerwood D. M. Petry N. M. (2007). Psychological factors that promote and inhibit pathological gambling. *Cogn. Behav. Practice* 14 208–217. 10.1016/j.cbpra.2006.02.005

[B25] Novus (2020). Allmänheten om spel 2020. Available online at: https://www.spelinspektionen.se/globalassets/dokument/statistik/enkatundersokning/allmanheten-om-spel-2020.pdf (accessed September 29, 2021).

[B26] Olason D. T. Hayer T. Meyer G. Brosowski T. (2017). Economic recession affects gambling participation but not problematic gambling: results from a population-based follow-up study. *Front. Psychol.* 8:1247. 10.3389/fpsyg.2017.01247 28790946 PMC5524821

[B27] Parke A. Griffiths M. Pattinson J. Keatley D. (2018). Age-related physical and psychological vulnerability as pathways to problem gambling in older adults. *J. Behav. Addict.* 7 137–145. 10.1556/2006.7.2018.18 29486572 PMC6035019

[B28] Pickering D. Spoelma M. J. Dawczyk A. Gainsbury S. M. Blaszczynski A. (2019). What does it mean to recover from a gambling disorder? Perspectives of gambling help service users. *Addict. Res. Theory* 28 132–143. 10.1080/16066359.2019.1601178

[B29] Price A. (2020). Online gambling in the midst of COVID-19: a nexus of mental health concerns, substance use and financial stress. *Int. J. Ment. Health Addict. [Online ahead of print]* 1–18. 10.1007/s11469-020-00366-1 32837444 PMC7357671

[B30] Public Health Agency (2019). *Results from Swelogs 2018.* Available online at: https://www.folkhalsomyndigheten.se/contentassets/e2f80df7971e4abfa615a5edcf460897/resultat-swelogs-2018-2019.pdf (accessed November 15, 2020).

[B31] Ramböll (2018). *SurveyXact [Online].Ramböll Management Consulting.* Available online at: https://www.surveyxact.se/ (accessed May 25, 2020).

[B32] Ronzitti S. Soldini E. Smith N. Bayston A. Clerici M. Bowden-Jones H. (2018). Are treatment outcomes determined by type of gambling? A UK study. *J. Gambl. Stud*. 34 987–997. 10.1007/s10899-018-9752-4 29383610

[B33] Sharman S. Murphy R. Turner J. J. D. Roberts A. (2019). Trends and patterns in UK treatment seeking gamblers: 2000-2015. *Addictive Behav.* 89 51–56. 10.1016/j.addbeh.2018.09.009 30248548

[B34] Sharman S. Roberts A. Bowden-Jones H. Strang J. (2021). Gambling in COVID-19 Lockdown in the UK: depression, stress, and anxiety. *Front. Psychiatry* 12:621497. 10.3389/fpsyt.2021.621497 33569018 PMC7868396

[B35] Sischka P. E. Décieux J. P. Mergener A. Neufang K. M. Schmidt A. F. (2020). The impact of forced answering and reactance on answering behavior in online surveys. *Soc. Sci. Comput. Rev.* 10.1177/0894439320907067

[B36] Velotti P. Rogier G. Beomonte Zobel S. Billieux J. (2021). Association between gambling disorder and emotion (dys)regulation: a systematic review and meta-analysis. *Clin. Psychol. Rev.* 87:102037. 10.1016/j.cpr.2021.102037 34022642

[B37] Wall H. Berman A. H. Jayaram-Lindström N. Hellner C. Rosendahl I. (2020). Gambler clusters and problem gambling severity: a cluster analysis of Swedish gamblers accessing an online problem gambling screener. *Psychol. Addict. Behav*. 35 102–112. 32614206 10.1037/adb0000674

[B38] Wardle H. Donnachie C. Critchlow N. Brown A. Bunn C. Dobbie F. (2021). The impact of the initial Covid-19 lockdown upon regular sports bettors in Britain: findings from a cross-sectional online study. *Addict. Behav.* 118:106876. 10.1016/j.addbeh.2021.106876 33647707 PMC9757982

[B39] Williams R. J. Volberg R. A. (2014). The classification accuracy of four problem gambling assessment instruments in population research. *Int. Gambl. Stud.* 14 15–28. 10.1080/14459795.2013.839731

[B40] Wood R. T. Williams R. J. (2007). ‘How much money do you spend on gambling?’ The comparative validity of question wordings used to assess gambling expenditure. *Int. J. Soc. Res. Methodol.: Theory Practice* 10 63–77. 10.1080/13645570701211209

[B41] Wynne H. Ferris J. (2001). *The Canadian Problem Gambling Index: Final Report.* Ottawa: Canadian Centre on Substance Abuse (CCSA).

