# Supplementary material for: A Longitudinal Study of Gambling Behaviors During the COVID-19 Pandemic in Sweden
Source: Front Psychol. 2021 Oct 14;12:708037. doi: 10.3389/fpsyg.2021.708037 (PMC8552012; doi:10.3389/fpsyg.2021.708037)
Supplement: Supplementary file 1 [file Table_1.DOCX]

**Supplementary material**

**Supplementary Table 1.** **Game types (n, %) and median gambling expenditures in SEK per game type***

| Game type | Pre-pandemic  (n=283) | | First wave  (n=267) | Second wave  (n=137) |
| --- | --- | --- | --- | --- |
| Online casino: slots | 96 (33.9)  1,500 | 93 (34.8)  3,000 | | 42 (30.7)  2,750 |
| Online casino: table games | 19 (6.7)  1,000 | 11 (4.1)  3,000 | | 8 (5.8) |
| Online casino: live casino | 31 (11.0) 1,500 | 24 (9.0)  1,250 | | 11 (8.0)  1,000 |
| Land-based casino games  in a casino with international rules | 10 (3.5)  3,000 | 3 (1.1) | | 1 (0.7) |
| Table- or card games in a restaurant | 6 (2.1) | 3 (1.1) | | 1 (0.7) |
| EGMs | 13 (4.6)  600 | 14 (5.2)  2,500 | | 3 (2.2) |
| Online sports betting, odds games | 79 (27.9)  1,000 | 50 (18.7)  1,000 | | 48 (35.0)  1,000 |
| Online sports betting, live betting | 50 (17.7)  1,000 | 30 (11.2)  1,000 | | 26 (19.0)  500 |
| Land-based sports betting | 16 (5.7)  350 | 6 (2.3) | | 3 (2.2) |
| Online poker | 41 (14.5)  2,000 | 35 (13.1)  1,500 | | 20 (14.6)  1,000 |
| Poker at a land-based casino  with international rules | 13 (4.6)  6,000 | 1 (0.4) | | 1 (0.7) |
| Land-based horse betting | 15 (5.3)  300 | 13 (4.9)  200 | | 5 (3.7) |
| Horse betting at the track | 6 (2.1) | 1 (0.4) | | 1 (0.7) |
| Online horse betting | 41 (14.5)  2,250 | 44 (16.5)  900 | | 20 (14.6)  1,000 |
| Land-based bingo | 7 (2.5) | 2 (0.8) | | 3 (2.2) |
| Online bingo | 12 (4.2)  90 | 11 (4.1)  250 | | 5 (3.7) |
| Land-based scratch tickets | 42 (14.8)  80 | 23 (8.7)  90 | | 14 (10.2)  110 |
| Online scratch tickets | 19 (6.7)  200 | 20 (7.5)  100 | | 16 (11.7)  100 |
| Subscription lotteries  (on weekly or monthly basis) | 11 (3.9)  110 | 9 (3.4) | | 7 (5.1) |
| Land-based number games | 12 (4.2)  200 | 10 (3.8)  150 | | 5 (3.7) |
| Online number games | 39 (13.8)  Info missing | 38 (14.2)  200 | | 20 (14.6)  400 |
| Did not gamble previous month | 79 (27.9) | 84 (31.5) | | 37 (27.0) |
| *Number of games played, mean (sd)* | 2.04 (2.33) | 1.65 (2.07) | | 1.90 (2.33) |

** Median gambling expenditures are presented for game types reported by 10 or more participants.*
